# Preparing the Long-Term Care Sector for Future Health Crises: A Set of Practical Pandemic Management Staffing Strategies

**DOI:** 10.3390/ijerph23040497

**Published:** 2026-04-14

**Authors:** Ibrahim Abughori, Houssem Eddine Ben-Ahmed, Megan Kaulius, Maura MacPhee, David Keselman, Lara Croll, Ramtin Hakimjavadi, Alison Phinney, Farinaz Havaei

**Affiliations:** 1 School of Nursing, University of British Columbia, Vancouver, BC V6T 1Z1, Canada; 2 Canadian Health Workforce Network, University of Ottawa, Ottawa, ON K1N 6N5, Canada; 3 Louis Brier Home and Hospital, Vancouver, BC V6M 1W9, Canada; 4 British Columbia Care Providers Association, Burnaby, BC V5H 4M2, Canada; 5 School of Medicine, University of Ottawa, Ottawa, ON K1H 8M5, Canada

**Keywords:** long-term care, pandemic management, healthcare delivery, healthcare workers, knowledge translation

## Abstract

**Highlights:**

**Public health relevance—How does this work relate to a public health issue?**

- The COVID-19 pandemic magnified and exacerbated already existing health inequities.
- Utilizing learnings from pandemic responses to COVID-19 could help mitigate further health inequities in the face of future crises.

**Public health significance—Why is this work of significance to public health?**

- Long-term care in Canada was a sector that was disproportionately affected by COVID-19, providing an opportunity for learning and for mitigating health disparities in future pandemics.
- This project employed an integrated knowledge translation (iKT) approach utilizing expert and lived experience to co-create best practices for managing future pandemics/epidemics in the LTC sector, strengthen this sector, and protect it against future health crises.

**Public health implications—What are the key implications or messages for practitioners, policy makers and/or researchers in public health?**

- Investing in current recruitment and retention strategies for the LTC workforce, standardizing evaluations of staffing adequacy and resident outcomes, engaging in contingency planning, and utilizing volunteers as appropriate were identified as key steps.
- This unique, iKT-driven approach to co-create suggested actions for staffing in the LTC sector provides rich, invaluable information; this approach could be utilized in other sectors to decrease health inequities fueled by the COVID-19 pandemic.

**Abstract:**

Utilizing an integrated knowledge translation framework (iKT), the purpose of this study was to identify best practices for long-term care (LTC) staffing in British Columbia, Canada, based on learnings from the COVID-19 pandemic. Through multiple data sources, including an electronic survey provided to LTC operators and knowledge-generation forums held with LTC community members, four staffing recommendations were created. Our major findings emphasize how the pandemic exposed and further exacerbated LTC workforce shortcomings and provide rich, contextual information to help create efficacious and practical outcomes and enhance public health. Our recommendations include conducting contingency planning for potential crises, increasing the use of volunteers, implementing recruitment and retention strategies for the LTC workforce, and standardizing evaluations of staffing adequacy and resident outcomes. These investments can serve to strengthen LTC currently and to protect against potential future health crises. This project highlights how lived experience can be utilized to address health inequities and bolster public health outcomes.

## 1. Introduction

The COVID-19 outbreak, declared a global pandemic in March 2020, brought to light long-standing inequities within Canada’s long-term care (LTC) sector [1,2]. Health inequities—defined by the World Health Organization as avoidable and unjust differences in health outcomes and access to care [3]—were tragically magnified during this crisis. While older adults were disproportionately impacted by COVID-19 globally, the effects were particularly devastating within LTC homes. In the early stages of the pandemic, LTC residents in Organization for Economic Co-Operation and Development (OECD) countries reported higher COVID-19-related death rates compared to community-living older adults, and Canada was among the hardest hit [4]. Despite having one of the lowest overall mortality rates among OECD nations, Canada saw nearly 70% of its COVID-19 deaths occur in LTC homes, far surpassing the OECD average of 41% [5]. These elevated death rates were attributed not only to the COVID-19 pandemic but also to persistent structural deficiencies in Canada’s LTC system that made it under-prepared for managing the COVID-19 pandemic [1,2]. While tragic, these systemic weaknesses provide learning opportunities highlighting how to mitigate such shortcomings in the future. Further, there is a marked paucity of guidelines for LTC sector leaders regarding future pandemic preparedness and management. The purpose of this study was to co-create best pandemic management staffing practices for the LTC sector using integrated knowledge translation (iKT) methods that captured the perspectives of diverse LTC experts, including LTC providers, leaders, managers, residents, and family caregivers [1,6]. These recommendations can help guide LTC leaders and operators with more effective management of future pandemics in this sector and highlight how lived experience can be integrated into public health matters.

### 1.1. Long-Term Care Sector in Canada: Residents and Staff

The LTC sector in Canada plays a critical role in supporting older adults who require continuous care due to aging, chronic illness, or cognitive decline. The resident population in Canadian LTC homes is largely composed of older individuals living with complex medical conditions, including dementia, frailty, and multiple chronic illnesses. Approximately 63% of LTC residents identify as female, slightly over half (51%) are 85 years of age or older, and nearly two-thirds of the population experience moderate to severe cognitive impairments [7].

In Canada, as much as 90% of direct hands-on care is provided by unregulated providers known as healthcare aides (HCAs) [1,8]. The remaining 10% of direct care is provided by regulated nurses; most frequently by licensed practical nurses (LPNs) and less frequently by registered nurses (RNs) [1,8]. Evidence suggests the proportion of regulated nurses to unregulated HCAs has decreased, meaning there are fewer LPNs and RNs and increased numbers of HCAs for the same expected level of care compared to two decades ago in Western Canada [9]. This staffing shift has been linked to poorer care outcomes for LTC residents [10]. Longstanding issues in LTC were magnified by the COVID-19 pandemic, highlighting the urgent need for systemic reform to ensure safe, equitable, and high-quality care for some of Canada’s most vulnerable citizens.

### 1.2. Pandemic Management Policies and Practices

In an effort to contain the spread of COVID-19, the BC Public Health Office implemented a series of pandemic management policies and practices in LTC, including restrictions on visitors, pre-entry screening protocols, and the Single Site Order (SSO) limiting staff from working across multiple high-risk areas such as LTC homes [11,12,13,14]. While these measures were effective in slowing the spread of the virus [15,16], they had far-reaching unintended consequences, including delayed care and isolation for residents and burnout for LTC staff and leaders due to drastically heightened workloads [14]. The COVID-19 pandemic exposed and exacerbated critical weaknesses in LTC management [11,12,13,14], making this an important area of ongoing research.

Early in the pandemic, our team conducted a case study to evaluate the consequences of various LTC pandemic management strategies in British Columbia (BC) [13,17]. Subsequently, we conducted an iKT project with other BC LTC knowledge users, co-creating best practices for managing future pandemics/epidemics in the LTC sector.

## 2. Materials and Methods

In order to bridge the gap between academic knowledge generation and practical usability, this project utilized an iKT approach. As the terminology suggests, an iKT approach integrates knowledge translation through all stages of the research, collaborating with knowledge users (individuals who are likely to be influenced by research results to make decisions) [6] throughout the process. Such participatory research approaches can have a multitude of positive effects on the research process and associated outcomes, including enhancing sustainability and capacity building [18].

Guided by these principles, our research team collaborated with an advisory group comprising LTC sector experts, including government officials, provincial association representatives, nurses, residents and family members, and researchers. The advisory group was engaged throughout the research process and collaboratively aided in outlining study methods and procedures, including survey development, recruitment, data collection, data analysis and interpretation, and knowledge dissemination. During the first six months of the project, the advisory group met monthly to support the project’s takeoff; in the second six months, meetings were held bimonthly to guide ongoing data collection and analysis.

The project took place over three phases that investigated seven overarching pandemic management practices and policy areas, including (1) infection screening, (2) visitation, (3) staffing, (4) infection prevention and control procedures, (5) communication, (6) physical layout, and (7) leadership and organizational support. This study reports on data pertaining to staffing strategies. Throughout the iKT process, LTC operators and staff identified staffing shortages and subsequent heavy workloads as the most concerning unintended consequences of COVID-19 and the most impactful factor of the pandemic management strategies. Descriptions for the other management strategies are in Appendix A.

In Phase I, an electronic survey was sent via Qualtrics to LTC operators who were purposefully identified by the research advisory group. The survey asked respondents to identify their LTC home characteristics (Table 1) and rate 34 different strategies based on the seven key pandemic management areas. A total of 97 LTC operators across BC’s six health authorities were contacted in August 2021 and invited to participate; a total of 19 individuals completed the survey (21% response rate). Descriptive statistics were used to analyze the quantitative data.

Phase II involved the convening of LTC community members across BC’s health authorities. In order to encourage maximum participation congruent with equity, diversity, and inclusion principles, inclusion/exclusion criteria were not communicated beyond specifying that interested participants must play a role in the LTC community (e.g., as an LTC operator, staff member, resident, or family member). A total of four virtual discussion forums were held between August and October 2021 for four of the five BC health authorities responsible for delivering healthcare services within their specific geographic regions. All participants in the focus groups were fluent in English, which ensured effective communication and facilitated the analysis of data. Table 2 shows participant roles at each virtual discussion forum.

A conversation guide was developed to help facilitate the knowledge generation forums and gather rich, detailed, and authentic insights. The guide included a list of open-ended questions and follow-up questions related to the overall research goals for the two moderators (FH, IA) to cover during the discussion forums. The guide was reviewed and revised based on feedback from the advisory group to ensure that questions were clearly and appropriately worded. Some of these questions were modified and reformulated to increase interactions and uncover deeper insights into participants’ experiences of the pandemic, all of which resulted in the generation of new ideas and a better understanding of the complex, often contradictory, viewpoints. A complete copy of the conversation guide is included in Appendix B.

The group discussions ranged from 90 to 120 min. Each Phase II forum began with an overview of the Phase I survey findings, followed by a moderated discussion and dialogue with forum participants about the effectiveness, implementation, and uptake of pandemic management strategies across LTC homes affiliated with each regional health authority. Input from the forum participants was used to develop a series of recommendations on best pandemic management strategies in LTC homes. After conducting four virtual discussion forums, the researchers (FH, IA) determined that the information gathered was sufficient and that additional sessions would have provided no new insights.

Phase III included a three-hour final virtual debrief in February 2022, with LTC members from all five regional health authorities and the research advisory group. During the final virtual debrief, the research team provided an overview of the project findings, shared the Phase II recommendations, and facilitated consensus-building on best pandemic management strategies across BC LTC homes.

## 3. Data Analysis

Quantitative data were analysed descriptively using IBM SPSS Statistics version 31 (IBM, Armonk, NY, USA). Qualitative data were analysed using content analysis [19]. Coding was conducted using pre-defined codes, including the pandemic management areas, and was focused primarily on identifying patterns, themes and trends related to the impact of the pandemic and its related public health measures. To ensure consistency in coding, two members of the research team with expertise in coding qualitative data (FH, IA) coded the transcripts independently. A codebook was then created and included a list of all identified codes, their definitions, and example quotes from the transcripts of the focus groups. Research team and advisory group members met regularly to review the codes, negotiate a common understanding of the codebook, analyze the results, make interpretations, and draw conclusions. Disagreements among the team (occurring very minimally) were resolved through open discussion and reviewing the project goals and objectives until a consensus was reached. Research team and advisory group members also met regularly to analyze the results, make interpretations, draw conclusions, and finally, propose recommendations for addressing staffing challenges in a pandemic.

## 4. Results

### 4.1. Phase I: LTC Operator Survey

As mentioned above, LTC operators were most concerned with pandemic management staffing strategies. Table 3 provides an overview of the operator survey responses pertaining to staffing strategies during COVID-19.

### 4.2. Phases II and III: Virtual Forums

The virtual forums reinforced the importance of effective pandemic/epidemic staffing strategies. As one participant stated, “Staffing levels prior to COVID-19 were already challenged with not being able to hire adequate, qualified staff. Empty lines and positions everywhere.” (LTC Staff Member).

Participants also expressed a multitude of concerns specific to the SSO, which prevented certain LTC providers from employment in more than one LTC home. There was a strong sense that the SSO exacerbated LTC staffing shortages (e.g., prohibiting or limiting casual staff from working in LTC homes, reducing staff’s choices of location to work, etc.). One LTC operator commented:

“Even with updated staffing plans and plans for increased staffing, due to single-site orders and a lack of staff…in LTCs in general, it was difficult to [get] staff to where we wanted and needed to be. During an outbreak, it was nearly impossible—people completely refused to work at times because we were in outbreak.”

LTC operators and staff also expressed concern about the lack of human and non-human resources to appropriately implement the SSO. For example, LTC homes were tasked with tracking where each staff member worked without being provided additional support to effectively complete this undertaking. Finally, LTC operators and staff identified another key limitation of the SSO as its operationalization and management by health authorities with no or only limited involvement and input from LTC operators and staff.

LTC operators also expressed concern about LTC working conditions and their impact on widening the supply and demand gap in the LTC workforce. Illustrated by one LTC operator, “I think we need to consider the fact is that long term care has been under-funded, under-resourced, under-staffed for a long, long time.”

**Figure 1:**
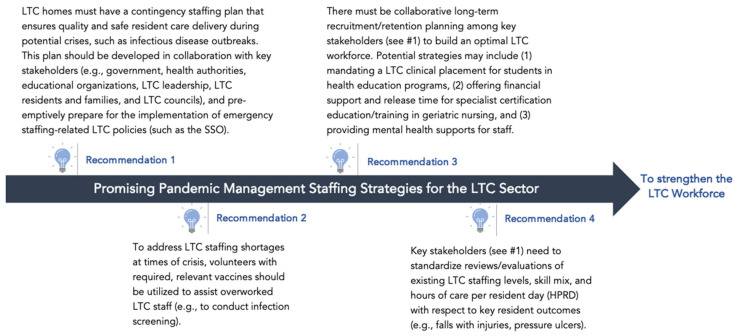
Recommendations for Addressing Staffing Challenges in a Pandemic.

Finally, LTC operators also shared concerns about the impact of LTC staffing shortages on the mental health of existing LTC staff and leaders and, subsequently, on quality and safe resident care delivery. LTC operators were most significantly concerned about the mental health of the newly graduated health workforce and novice leaders in the sector. As stated by one participant, “A newly graduated registered nurse won’t survive long in long-term care without support and mentorship. It is a different kind of environment.” See Figure 1 for recommendations based on these findings.

## 5. Discussion

Our iKT project aimed to co-create a series of best practices in BC LTC staffing based on knowledge gained through the COVID-19 pandemic [1]. Key post-pandemic recommendations focused on collaborative contingency staffing for potential crises, enhanced recruitment and retention strategies for the LTC workforce, and standardized evaluations of LTC staffing.

Contingency planning for LTC staffing depends on the co-development of supportive human resource policies that include LTC leadership and health emergency management experts at regional and provincial levels [20]. Post-COVID-19, lack of communication and coordination among LTC leaders and health authorities was associated with delays in receiving necessary resources and supports [21]. A plan co-created by LTC key stakeholders (e.g., LTC leadership, staff, residents, and family members alongside government and health authority leadership) should be made to pre-emptively take action in future public health emergencies [11,22,23,24]. A previous report exploring the BC Ministry of Health response to COVID-19 in the LTC sector highlights gaps in coordination, role clarity, and consistency across health authorities and LTC providers, which contributed to variability in staffing support and operational responses [11]. It also underscored how pre-existing workforce shortages, compounded by policies such as the single-site order, exposed the absence of a coordinated, system-wide approach to staffing during emergencies [11]. These findings directly support our recommendation, reinforcing the need for collaborative workforce planning mechanisms to enable effective and equitable emergency response in LTC.

LTC staffing for emergencies is currently hampered by significant national health human resource shortages. The 2025 Canadian Institute for Health Information (CIHI) report highlights that there are fewer healthcare workers employed in the LTC sector compared to before the pandemic [25]. Therefore, creative staffing strategies need to be employed, such as through the use of volunteers. However, while volunteers could support non-clinical tasks, their onboarding, training, and responsibilities would require careful planning.

Longer-term recruitment and retention staffing strategies are needed to address negative stereotypes associated with LTC that make this sector unattractive to healthcare workers. The need for improved career pipelines in LTC is evident. Some options for increasing interest in LTC careers include collaborations between LTC homes and academic programs where faculty introduce students to geriatric nursing as a specialty practice [26], or implementing residency programs that support the transition of new nurses into LTC [22]. In terms of staff retention, participants in our study raised the importance of protecting LTC staff and leadership mental health. One promising approach, borrowed from acute care, is the Schwartz Rounds Program. This is a peer-support program that offers staff a safe space to express their emotional and psychological challenges and share their experiences through a storytelling approach [23,24,27]. Research has shown the benefits of this program in increasing emotional resilience, instilling compassion, and supporting the well-being of healthcare workers [23,24,28,29].

Recent Canadian data from the LTC sector point to a clear and concerning contraction of the workforce, particularly among regulated nurses, alongside growing strain in the unregulated workforce. By 2023, only 13.6% of Canada’s health workforce was employed primarily in LTC—lower than pre-pandemic levels—suggesting a broader shift away from the sector [25]. This decline is most pronounced among RNs, whose numbers in LTC decreased by 42.5% between 2014 and 2023 (from 548 to 315 per reporting unit) [25]. Although LPNs and RPNs continue to comprise the largest segments of the regulated workforce in LTC, they have also experienced modest declines since 2021 (−6.1% and −2.1%, respectively) [25]. These trends are particularly concerning in the context of increasing resident complexity, which has risen by approximately 4.5%, thereby intensifying demand for professional nursing care [25]. This growing mismatch is further underscored by a robust body of evidence demonstrating that higher levels of regulated nurse staffing are associated with improved resident outcomes [10,30]. Taken together, these findings highlight a need to strengthen recruitment and retention strategies targeting the regulated nursing workforce in LTC.

Finally, evaluating LTC sector performance requires the use of standardized data to ensure consistent, comparable, and evidence-based assessments over time. CIHI provides a valuable source of such data, enabling sector-wide monitoring and benchmarking. Using CIHI data more regularly to examine trends in staffing (e.g., actual hours per resident per day) and resident outcomes (e.g., falls, the use of antipsychotic medication) could provide valuable information on LTC sector functioning. As noted by CIHI, data collection on HCAs is inconsistent across Canada [25] and requires standardization to fully capture LTC sector functioning. This is particularly important as an overwhelming majority of care is delivered by HCAs in Canadian LTC homes. Complementing these quantitative measures, qualitative insights should also be gathered through informal or formal methods of data collection, such as conversations and focus groups with residents, families, and care providers, which would offer an essential, human perspective on care quality and the lived experience in LTC settings. Without regular data monitoring, proactive staffing planning is not possible [25]. This recommendation is consistent with a growing body of literature linking staffing levels and skill mix to resident outcomes, while also highlighting ongoing challenges related to inconsistent data collection and the need to complement standardized quantitative indicators with qualitative insights [10,30]. Together, these findings suggest that while standardized indicators are increasingly being used in Canada, gaps in data completeness and comparability continue to limit their full utility, reinforcing the need for more consistent and standardized approaches to evaluating both staffing adequacy and resident outcomes [31].

Overall, these suggestions provide rich data based on lived experience that pave the way for decreased health inequities and provide a method for integrating lived experience into public health decisions.

### Strengths and Limitations

A limitation of note is that the quantitative data gathered from LTC operators via electronic survey yielded a low response rate, potentially influenced by the fact that the study took place during the early stages of the COVID-19 pandemic. The participants who did respond may represent a subset of operators (e.g., those with enough organizational support to participate in knowledge translation efforts), affecting the outcomes presented in this paper. Relatedly, the findings of this knowledge translation project are contextual, and it is unclear how generalizable the learnings from the COVID-19 pandemic could be to future health crises. Despite these limitations, this study has a number of important strengths to highlight. First, this iKT study sought to involve LTC community members and experts through all phases of our project. Grounded in participants’ lived experiences, participatory research approaches are associated with enhanced potential for effecting real-world change [18]. Additionally, our study utilized a combination of quantitative and qualitative data in our research approach, allowing for a more robust exploration of the effects of the COVID-19 pandemic on staffing in long term care. While we are not able to generalize these findings, the contextual and rich nature of our data is a noteworthy strength. Building upon our findings with more robust qualitative and quantitative research would be a warranted area of future work.

## 6. Conclusions

The COVID-19 pandemic revealed the inadequate preparedness of the LTC sector to meet the challenges of a crisis. Direct knowledge gained from the front lines of the LTC sector during the COVID-19 crisis provides rich information to help create effective, impactful, and practical outcomes. The recommendations garnered through this process outline the importance of contingency planning for potential crises, implementing recruitment and retention strategies for the LTC workforce, and standardizing evaluations of staffing adequacy and resident outcomes. Strategic investments and workforce planning are needed now to protect this vital healthcare sector. The recommendations of best practices, derived through the iKT approach, can inform ongoing LTC staffing efforts for potential crises and eventual creation of a viable LTC workforce. Further, the unique iKT approach of our project provides a roadmap for the inclusion of lived experience to address health inequities and bolster public health outcomes.

## Figures and Tables

**Table 1:** LTC Home Information (Phase I).

Variable	Number (Percentage)
Funding Model	
Publicly owned (health authority)	4 (21.1%)
Private for-profit	8 (42.1%)
Private not-for-profit	7 (36.8%)
Accreditation	
Yes	13 (68.4%)
No	6 (31.6%)
Number of Beds	
Up to 99	9 (47.4%)
100–199	6 (31.6%)
200+	4 (21.1%)
Age of LTC Home	
0–14	4 (21.1%)
15–29	5 (26.3%)
30–44	5 (26.3%)
45+	5 (26.3%)
Experience of a COVID-19 Outbreak	
Yes	11 (57.9%)
No	8 (42.1%)
Health Authority	
Interior Health Authority	4 (15.4%)
Fraser Health Authority	9 (34.6%)
Vancouver Coastal Health Authority	5 (19.2%)
Vancouver Island Health Authority	1 (3.8%)

**Table 2:** Phase II and III discussion forum participants’ roles.

Participants	Forum 1: Interior Health Authority	Forum 2: Vancouver Island Health Authority	Forum 3: Fraser Health Authority	Forum 4: Vancouver Coastal Health Authority	Final Debrief Forum
Administrators	2	5	1	1	7
Nurses	1	2	1	1	0
Residents	1	1	1	0	2
Families	3	4	5	1	2
Other	5	4	5	6	10
Total	12	16	13	9	21

Note: The discussion forum with LTC members from the Northern Health Authority was cancelled due to a lack of participation in the midst of a significant wildfire in the region at the time of the study.

**Table 3:** Pandemic Management Staffing Strategies Survey Results.

Staffing Strategies	N (%)
Updated staffing plan in place	
Always implemented	15 (83.3)
Important for mitigating risk of spreading COVID-19	16 (88.9)
Important for the physical and mental health of staff	17 (94.5)
Important for the physical and mental health of residents	15 (83.3)
Important for the physical and mental health of families	14 (77.7)
Increasing staffing levels beyond baseline	
Always implemented	14 (82.4)
Important for mitigating risk of spreading COVID-19	18 (100)
Important for the physical and mental health of staff	18 (100)
Important for the physical and mental health of residents	16 (88.9)
Important for the physical and mental health of families	16 (88.9)
Contingency staffing plan in place	
Always implemented	15 (88.2)
Important for mitigating risk of spreading COVID-19	18 (100)
Important for the physical and mental health of staff	18 (100)
Important for the physical and mental health of residents	16 (88.9)
Important for the physical and mental health of families	15 (83.3)
Flexible and responsive sick policy in place	
Always implemented	14 (82.4)
Important for mitigating risk of spreading COVID-19	17 (94.4)
Important for the physical and mental health of staff	17 (94.4)
Important for the physical and mental health of residents	15 (83.3)
Important for the physical and mental health of families	12 (66.6)
Organizational support in place for staff impacted by SSO	
Always implemented	12 (70.6)
Important for mitigating risk of spreading COVID-19	18 (100)
Important for the physical and mental health of staff	18 (100)
Important for the physical and mental health of residents	14 (77.7)
Important for the physical and mental health of families	12 (66.7)
Interventions, tools or resources in place to support staff with rising workloads	
Always implemented	12 (70.6)
Important for mitigating risk of spreading COVID-19	17 (94.4)
Important for the physical and mental health of staff	18 (100)
Important for the physical and mental health of residents	16 (88.9)
Important for the physical and mental health of families	14 (77.7)

Note: LTC operators were asked to what extent the strategy was implemented (response options included yes, no, or to some extent, with the option for the respondent to describe). The operators were then asked to indicate how important the strategy was in (a) mitigating the risk of spreading COVID-19, and (b) protecting the health and safety of LTC (i) staff, (ii) residents, and (iii) families. Responses were indicated through a four-point scale from “not at all important” to “extremely important”. In this table, important includes “somewhat important” and “extremely important” response options; SSO = Single Site Order.

## Data Availability

The data presented in this study are available on request from the corresponding author.

## Appendix A. Key Pandemic Management Areas

**Area**	**Description**
Screening	Practices and procedures relating to COVID-19 screening of LTC home visitors prior to entry.
Visitation	The policy restricting visitors from visiting their loved ones residing in LTC homes during COVID-19.
Staffing	Practices and strategies relating to ensuring adequate staffing levels and appropriate skill mix to meet the needs of LTC residents in the context of COVID-19.
Infection Prevention and Control Procedures	Practices and procedures relating to preventing the spread of COVID-19 and ensuring the safety of LTC residents and staff.
Communication	Practices and procedures relating to the exchange of important information, updates and policies with LTC homes, their staff, residents and their families in the context of COVID-19.
Physical Layout	The design/re-design of the physical environment consistent with infection prevention and control guidelines and to ensure the safety of LTC residents and staff in the context of COVID-19.
Leadership and Organizational Support	Practices and strategies adopted by LTC homes and their leadership to support the health and safety of LTC residents and staff in the context of COVID-19.

## Appendix B. Forum Conversation Guide

**Domain**	**Key Questions & Prompts**
**Screening, Visitation & Family Engagement**	- How were visitation policies implemented in your setting? − What worked well in maintaining family connection? − What challenges did you face (e.g., clarity, consistency)? - How were “essential visitors” defined and operationalized? − Were there inconsistencies across sites or staff? - What would you do differently in future outbreaks? − How can we better balance safety with emotional well-being?
**Staffing & Workforce Capacity**	- How did staffing levels and skill mix change during the pandemic? − What were the biggest staffing challenges? - What strategies were used to maintain staffing capacity? − Were contingency plans effective? - How did staffing pressures impact care delivery and staff well-being? − What contributed to burnout or retention challenges?
**Infection Prevention & Control (IPC)**	- How prepared was your organization for IPC at the onset of the pandemic? − What worked well in implementing IPC measures? - What challenges did you encounter? − Training, staffing, or resource gaps? - What system or infrastructure gaps became evident? − Access to PPE, IPC expertise, or guidance?
**Communication & Information Flow**	- How were changes in policies and guidelines communicated? − What worked well with staff and families? - Where did breakdowns or delays occur? − Were messages clear and consistent? - How can communication be improved in future crises? − What channels and approaches are most effective?
**Physical Environment & Infrastructure**	- How did the physical layout of your LTC home impact infection control? − What limitations did you face (e.g., shared rooms, space)? - What changes were made—or could be made—to improve safety? − How can environments better support cohorting or distancing?
**Leadership & Organizational Support**	- How did leadership support staff and residents during the pandemic? − What leadership practices were most effective? - What gaps existed in leadership preparedness or support? − Were leaders adequately trained for crisis response? - What supports are needed for leaders moving forward?
**Innovations & Promising Practices**	- What innovative strategies emerged during the pandemic? − Which practices were most impactful? - What should be sustained moving forward? − What belongs in a future “playbook”?

## References

[1] Estabrooks C.A., Straus S.E., Flood C.M., Keefe J., Armstrong P., Donner G.J., Boscart V., Ducharme F., Silvius J.L., Wolfson M.C. (2020). Restoring Trust: COVID-19 and the Future of Long-Term Care in Canada. Facets.

[2] Hsu A.T., Lane N., Sinha S.K., Dunning J., Dhuper M., Kahiel Z., Sveistrup H. (2020). Understanding the Impact of COVID-19 on Residents of Canada’s Long-Term Care Homes—Ongoing Challenges and Policy Responses. Int. Long-Term Care Policy Netw..

[3] World Health Organization (2021). Health Equity and Its’ Determinants.

[4] Sepulveda E.R., Stall N.M., Sinha S.K. (2020). A Comparison of COVID-19 Mortality Rates Among Long-Term Care Residents in 12 OECD Countries. J. Am. Med. Dir. Assoc..

[5] Canadian Institute for Health Information (2021). The Impact of COVID-19 on Long-Term Care in Canada: Focus on the First 6 Months.

[6] Canadian Institutes for Health Research (2012). Guide to Knowledge Translation Planning at CIHR: Integrated and End-of-Grant Approaches.

[7] Canadian Institute for Health Information (2024). Profile of Residents in Residential and Hospital-Based Continuing Care, 2023–2024.

[8] Estabrooks C.A., Squires J.E., Carleton H.L., Cummings G.G., Norton P.G. (2015). Who Is Looking After Mom and Dad? Unregulated Workers in Canadian Long-Term Care Homes. Can. J. Aging.

[9] Squires J.E., Baumbusch J., Demery Varin M., MacDonald I., Chamberlain S., Boström A.-M., Thompson G., Cummings G., Estabrooks C.A. (2019). A Profile of Regulated Nurses Employed in Canadian Long-Term Care Facilities. Can. J. Aging.

[10] Clemens S., Wodchis W., McGilton K., McGrail K., McMahon M. (2021). The Relationship between Quality and Staffing in Long-Term Care: A Systematic Review of the Literature 2008–2020. Int. J. Nurs. Stud..

[11] Ernst & Young BC Ministry of Health (2020). Long-Term Care COVID-19 Response Review.

[12] Liu M., Maxwell C.J., Armstrong P., Schwandt M., Moser A., McGregor M.J., Bronskill S.E., Dhalla I.A. (2020). COVID-19 in Long-Term Care Homes in Ontario and British Columbia. Can. Med. Assoc. J..

[13] Havaei F., Abughori I., Mao Y., Staempfli S., Ma A., MacPhee M., Phinney A., Keselman D., Tisdelle L., Galazka D. (2022). The Impact of Pandemic Management Strategies on Staff Mental Health, Work Behaviours, and Resident Care in One Long-Term Care Facility in British Columbia: A Mixed Method Study. J. Long Term Care.

[14] Staempfli S., Havaei F., Phinney A., MacPhee M. (2022). Unintended Consequences of Pandemic Management Strategies on Residents and Family in One Long-Term Care Home in British Columbia: A Patient-Supported Qualitative Study. J. Innov. Aging.

[15] Brown K.A., Buchan S.A., Chan A.K., Costa A., Daneman N., Garber G., Hillmer M., Jones A., Johnson J.M., Kain D. (2024). Association between Delayed Outbreak Identification and SARS-CoV-2 Infection and Mortality among Long-Term Care Home Residents, Ontario, Canada, March to November 2020: A Cohort Study. Euro Surveill..

[16] Vijh R., Prairie J., Otterstatter M.C., Hu Y., Hayden A.S., Yau B., Daly P., Lysyshyn M., McKee G., Harding J. (2021). Evaluation of a Multisectoral Intervention to Mitigate the Risk of Severe Acute Respiratory Coronavirus Virus 2 (SARS-CoV-2) Transmission in Long-Term Care Facilities. Infect. Control Hosp. Epidemiol..

[17] Havaei F., MacPhee M., Keselman D., Staempfli S. (2021). Leading a Long-Term Care Facility through the COVID-19 Crisis: Successes, Barriers and Lessons Learned. Health Q..

[18] Jagosh J., Macaulay A.C., Pluye P., Salsberg J., Bush P.L., Henderson J., Sirett E., Wong G., Cargo M., Herbert C.P. (2012). Uncovering the Benefits of Participatory Research: Implications of a Realist Review for Health Research and Practice. Milbank Q..

[19] Hsieh H.-F., Shannon S.E. (2005). Three Approaches to Qualitative Content Analysis. Qual. Health Res..

[20] Peterson L.J., Dobbs D., June J., Dosa D.M., Hyer K. (2021). “You Just Forge Ahead”: The Continuing Challenges of Disaster Preparedness and Response in Long-Term Care. Innov. Aging.

[21] Siu H.Y.-H., Kristof L., Elston D., Hafid A., Mather F. (2020). A Cross-Sectional Survey Assessing the Preparedness of the Long-Term Care Sector to Respond to the COVID-19 Pandemic in Ontario, Canada. BMC Geriatr..

[22] Cadmus E., Salmond S.W., Hassler L.J., Black K., Bohnarczyk N. (2016). Creating a Long-Term Care New Nurse Residency Model. J. Contin. Educ. Nurs..

[23] McCarthy I., Taylor C., Leamy M., Reynolds E., Maben J. (2021). ‘We Needed to Talk about It’: The Experience of Sharing the Emotional Impact of Health Care Work as a Panellist in Schwartz Center Rounds^®^ in the UK. J. Health Serv. Res. Policy.

[24] Hofmeyer A., Taylor R., Kennedy K. (2020). Fostering Compassion and Reducing Burnout: How Can Health System Leaders Respond in the COVID-19 Pandemic and Beyond?. Nurse Educ. Today.

[25] Canadian Institute for Health Information (2025). Recent Staffing and Quality Indicator Trends in Canadian Long-Term Care.

[26] Naughton C., O’Shea K.L., Hayes N. (2019). Incentivising a Career in Older Adult Nursing: The Views of Student Nurses. Int. J. Older People Nurs..

[27] Ben-Ahmed H.E., Bourgeault I.L. (2023). Sustaining the Canadian Nursing Workforce: Targeted Evidence-Based Reactive Solutions in Response to the Ongoing Crisis. Can. J. Nurs. Leadersh..

[28] Golding L. (2024). Schwartz Rounds: Supporting the Emotional Wellbeing of Our Future Healthcare Workforce. Future Healthc. J..

[29] Maben J., Taylor C., Reynolds E., McCarthy I., Leamy M. (2021). Realist Evaluation of Schwartz Rounds^®^ for Enhancing the Delivery of Compassionate Healthcare: Understanding How They Work, for Whom, and in What Contexts. BMC Health Serv. Res..

[30] Yang B.K., Carter M.W., Trinkoff A.M., Nelson H.W. (2021). Nurse Staffing and Skill Mix Patterns in Relation to Resident Care Outcomes in US Nursing Homes. J. Am. Med. Dir. Assoc..

[31] Canadian Institute for Health Information (2026). Aging with Dignity: Using Data to Strengthen Long-Term Care for Canadians.

